# eNanoMapper: harnessing ontologies to enable data integration for nanomaterial risk assessment

**DOI:** 10.1186/s13326-015-0005-5

**Published:** 2015-03-21

**Authors:** Janna Hastings, Nina Jeliazkova, Gareth Owen, Georgia Tsiliki, Cristian R Munteanu, Christoph Steinbeck, Egon Willighagen

**Affiliations:** European Molecular Biology Laboratory – European Bioinformatics Institute (EMBL-EBI), Cambridge, United Kingdom; IdeaConsult Ltd., 4.A.Kanchev str., Sofia, Bulgaria; National Technical University of Athens (NTUA), Athens, Greece; Computer Science Faculty, University of A Coruña, A Coruña, Spain; Department of Bioinformatics – BiGCaT, NUTRIM, Maastricht University, Maastricht, Netherlands

**Keywords:** Nanomaterial, Safety, Ontology

## Abstract

Engineered nanomaterials (ENMs) are being developed to meet specific application needs in diverse domains across the engineering and biomedical sciences (e.g. drug delivery). However, accompanying the exciting proliferation of novel nanomaterials is a challenging race to understand and predict their possibly detrimental effects on human health and the environment. The eNanoMapper project (www.enanomapper.net) is creating a pan-European computational infrastructure for toxicological data management for ENMs, based on semantic web standards and ontologies. Here, we describe the development of the eNanoMapper ontology based on adopting and extending existing ontologies of relevance for the nanosafety domain. The resulting eNanoMapper ontology is available at http://purl.enanomapper.net/onto/enanomapper.owl. We aim to make the re-use of external ontology content seamless and thus we have developed a library to automate the extraction of subsets of ontology content and the assembly of the subsets into an integrated whole. The library is available (open source) at http://github.com/enanomapper/slimmer/. Finally, we give a comprehensive survey of the domain content and identify gap areas. ENM safety is at the boundary between engineering and the life sciences, and at the boundary between molecular granularity and bulk granularity. This creates challenges for the definition of key entities in the domain, which we also discuss.

## Background

Nanomaterials are materials in which the individual components are sized roughly in the 1-100 nanometer range in at least one dimension, although an exact definition is still being debated [1,2]. Particles in this size range display special properties having to do with their very large ratio of surface area to volume [3]. Natural nanomaterials include viral capsids and spider silk. Recent years have seen an explosion in the development of engineered nanomaterials (ENMs) aiming to exploit the special properties of these materials in various domains including biomedicine (e.g. as vehicles for drug delivery), optics and electronics [3].

Counterbalancing the many possible benefits of developed nanotechnology, nanoparticles also pose serious risks to human and environmental health [4]. Recognising these dangers, regulatory bodies are calling for systematic and thorough toxicological and safety investigations into ENMs with the objective of feeding knowledge into predictive tools which are able to assist researchers in designing safe nanomaterials. Evaluating and predicting the possible dangers of different nanomaterials requires assembling a wealth of information on those materials – the composition, shape and properties of the individual nanoparticles, their interactions with biological systems across different tissues and species, and their diffusion behaviour into the natural environment. These data are arising from different disciplines with highly heterogeneous requirements, methods, labelling and reporting practices. Regulatory descriptions of ENMs are not like those needed for nanoQSAR analyses. Safety requirements may also vary under different conditions, e.g. when developing vehicles for drug delivery in life-threatening diseases as compared to materials for use in the construction industry.

The eNanoMapper project (www.enanomapper.net) is creating a pan-European computational infrastructure for toxicological data management for ENMs, based on semantic web standards and ontologies. eNanoMapper aims to develop a comprehensive ontology and annotated database for the nanosafety domain to address the challenge of supporting the unified annotation of nanomaterials and their relevant biological properties, experimental model systems (e.g. cell lines), conditions, protocols, and data about their environmental impact. Rather than starting afresh, the developing ontology will build on existing work, integrating existing ontologies in a flexible pipeline. The establishment of a universal standardisation schema and infrastructure for nanomaterials safety assessment is a key project goal, which will support collaboration, integrated analysis, and discoveries from data organised within a knowledge-based framework.

In this paper, we survey the existing ontologies that were integrated into the unified eNanoMapper ontology, focusing on the challenges we experienced with the integration of diverse sources and our automated solution for seamless modular re-use of external content. Furthermore, we discuss challenges in the definition of key entities in the domain and give harmonised definitions for the core material and experimental entities in the domain.

## Problem

The eNanoMapper ontology covers the following broad content areas:

1. A categorisation of nanoparticle classes based on their properties, constituency and shape.
2. Physicochemical properties for ENM characterisation.
3. Biological characterisation that describes the ENM-specific interactions with, for example, proteins to form a corona.
4. Environmental characterisation.
5. Experimental design and encoding for experiments in which nanosafety is assessed.
6. The full nanomaterial lifecycle including manufacturing and environmental decay or accumulation.
7. Known safety information about ENMs.

Table 1 gives a summary of the ontologies that have been identified as already in part covering these domain areas. The selection of ontologies is motivated by the requirement that the ontologies be (a) *open*, that is, licensed for re-use without restriction other than attribution, (b) suitable for use in data annotation, i.e. using unique numeric identifiers and offering textual labels and definitions, and (c) be broadly mutually compatible (although with some provisos as we will discuss in the section on our re-use pipeline below).

**Table 1:** **A summary of the ontologies that have been identified as covering content areas of relevance for eNanoMapper**

**Ontology detail**	**Availability**	**Primary content and focus**
NanoParticle Ontology (NPO), [5]	http://bioportal.bioontology.org/ontologies/NPO	Nanomaterial types, properties and experiments
Chemical Entities of Biological Interest (ChEBI), [6]	http://www.ebi.ac.uk/chebi/	Chemical compounds, groups and roles, and nanomaterial types
Chemical information ontology (CHEMINF), [7]	http://code.google.com/p/semanticchemistry/	Chemical qualities and descriptors, both calculated and measured
Chemical Methods Ontology (CHMO)	http://purl.bioontology.org/ontology/CHMO	Chemical processes and experimental methods
Physico-Chemical Process Ontology (REX)	http: //www.obofoundry.org/cgi-bin/detail.cgi?id=rex	Chemical processes and experimental methods
Unit Ontology (UO), [8]	http://code.google.com/p/unit-ontology/	Units for measured or calculated quantities
Phenotype and Quality Ontology (PATO), [9]	https://code.google.com/p/pato/	Qualities and phenotypes
Ontology for Biomedical Investigations (OBI), [10]	http://obi-ontology.org/	Experiments and assays
BioAssay Ontology (BAO), [11]	http://bioassayontology.org/	Experiments and assays
Gene Ontology (GO), [12]	http://amigo.geneontology.org/	Molecular functions, biological processes and cellular components
Protein Ontology (PRO), [13]	http://pir.georgetown.edu/pro/pro.shtml	Proteins and protein complexes
Cell Ontology (CL), [14]	https://code.google.com/p/cell-ontology/	Cell types
Cell Culture Ontology (CCONT), [15]	http://purl.bioontology.org/ontology/CCONT	Cell lines
UBERON, [16]	http://uberon.org	Multi-species anatomy
Environment Ontology (ENVO), [17]	http://purl.bioontology.org/ontology/ENVO	Environments such as soil and sediment
Ontology of Adverse Events (OAE), [18]	http://purl.bioontology.org/ontology/OAE	Adverse events of a medical nature

These ontologies are described further in the Results section below. However, our initial “naïve” attempt to re-use these ontologies in their entirety in the development of the integrated eNanoMapper ontology ran into several challenges:

- Some content was duplicated across multiple different ontologies, resulting in multiple classes with different identifiers being included with the same label – including cases where classes with the same label were defined differently across the ontologies;
- Some classes which were multiply imported, i.e. following the recommended re-use policy, in the ontologies we imported, such as frequently used upper-level classes or unit classes, subsequently were found to have multiple copies of all associated annotations and axioms in the resulting composite ontology;
- It was difficult to reconcile the different upper level ontologies that were used in these ontologies, and in some cases even when the same upper level ontology was used (BFO), different versions of that upper level still caused incompatibilities;
- The presence of gaps in the imported content necessitates the manual annotation of content additions (which may later be submitted to various source ontologies). It was not easy to seamlessly add manual content to the imported ontologies without needing to re-create the manual content every time the source ontologies changed and were re-imported; and
- Some of the definitions of the classes we wanted to re-use were missing or not sufficiently clear for unambiguous re-use as part of an integrated whole.

Based on exact label matching only, the overlap between the ChEBI ontology and the NPO is 395. This is a small but nevertheless significant number of exactly shared labels. Most of these are groups, atoms or chemical classes that are included in NPO so as to support description of nanomaterial composition. Some, but not all, of these are cross-referenced to ChEBI via an additional annotation ‘dbXref’ in NPO. Other classes with overlapping labels derive from the fledgling nanoparticle classification that is included in ChEBI. For this branch of NPO, there are no cross-references annotated to ChEBI (and neither does ChEBI annotate cross-references to NPO). Some of the overlap arises from drug classes that are included in the NPO, e.g. thalidomide and tamoxifen, assumedly because the NPO was designed for cancer nanotechnology research and these are cancer drugs.

The OBO Foundry recommends collaboration to resolve overlap between neighbouring ontologies in situations such as these. A strategy that suggests always favouring one ontology over another is not possible, since for groups and chemical classes the ChEBI IDs are preferred, while for nanoparticle classes it is the NPO IDs.

Between BAO and NPO there are 37 overlapping labels. These include abstract classes such as ‘physical quality’, ‘shape’, ‘size’; and role classes such as ‘solvent’, ‘dihydrofolate reductase inhibitor’ and ‘fluorochrome’. Note that label sharing in itself is not a problem unless the IDs are different. If the MIREOT strategy is followed [19], the IDs and definitions will be exactly the same, which presents no problem for data annotation. This is the case for the bulk of the overlap between BAO and ChEBI, which with 696 shared labels would otherwise be very challenging to resolve.

Thus, in order to create a seamless and unified whole ontology, rather than importing the external ontologies in full we decided to create a pipeline for ontology re-use that enabled the import of *parts* of the external content, assembly of those parts beneath a slim upper level, and the use of those parts together with manually added content in a way that would be unaffected by regular updates of the source ontologies. This pipeline needed to be able to fit together imported ‘branches’, ‘leaves’ or diverse parts of external ontologies, and branches of manually annotated content, like jigsaw pieces together into a unified whole. Our strategy for managing duplication in the combined ontology resource is to systematically prune (i.e. remove) duplicated content as part of the import process for ontologies that are re-used. In each case, a primary provider for the content domain is selected. For example, for nanoparticle classification it is the NPO, for small molecules such as drugs it is ChEBI, for biological assays it is OBI etc. Duplicated content in other ontologies that are imported are then removed automatically by our pipeline.

In the next section we describe the pipeline we have created for this purpose and the way we have applied it to build up the eNanoMapper ontology from the component ontologies.

## Results

The eNanoMapper ontology is being developed in the Web Ontology Language (OWL), Version 2 [20]. One of the primary requirements on the source ontologies we chose to adopt is that they are available in OWL. However, as described above, in most cases we do not want to import the full external ontology. This may be because we only require a portion of the content, or because we need to exclude content that has already been imported from elsewhere thus would cause a duplicate if imported. A particularly challenging form of duplicated content for a complex application ontology such as eNanoMapper arises from multiply chained imports. When OWL imports the same content (e.g. ChEBI together with the portion of ChEBI that is already imported into the NPO), any associated metadata and axioms are duplicated. Thus, for core ontologies such as BFO that are imported by multiple other ontologies, the metadata are duplicated multiple times. This is expected behaviour in the context of OWL but presents a problem for complex import chains of ontologies as is needed for eNanoMapper.

For these reasons, we have created an ontology ‘slimming’ library which extracts subsets from existing ontologies according to predefined criteria. Subsets are then placed in a special folder (/external/) in the ontology development area, which is located on GitHub at http://github.com/enanomapper/ontologies. The subsets may be created through the identification of classes to extract, with the option to include their parents (paths to root) and/or children (paths to the leaves). Specific metadata and axioms may be identified for inclusion or exclusion. By default, all classes in the source ontology are excluded.

We are using Jenkins as a platform, similarly to the pipeline described in Mungall *et al.* [21], to enable continuous integration with logical consistency and coherence testing so as to ensure that the incorporation of updates from source ontologies does not lead to fragmentation and inconsistencies in the integrated whole. Our continuous build system is publicly available online at http://jenm.bigcat.maastrichtuniversity.nl/. We are performing multiple quality control tests on a regular basis in addition to the composite ontology consistency check, including checking for the presence of labels and definitions across all classes included in the resource.

**Figure 1:**
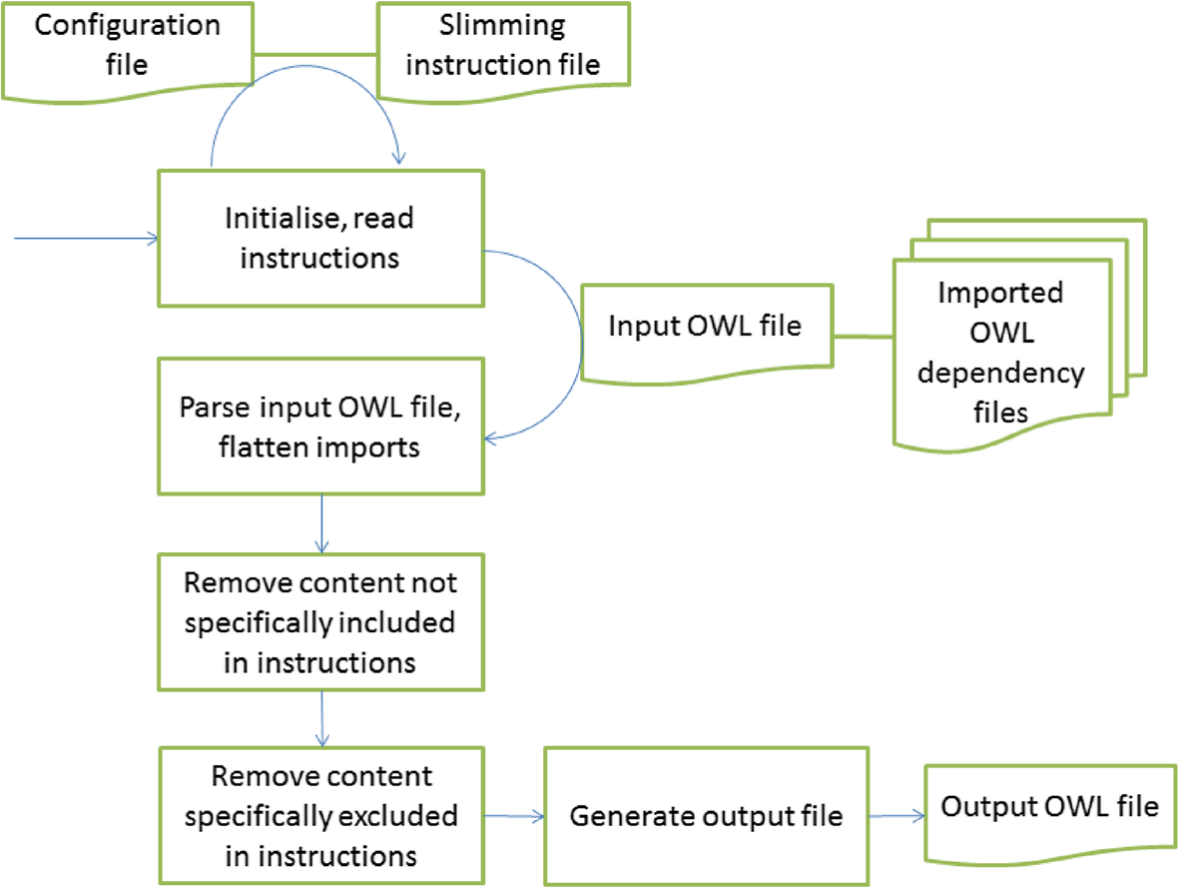
**The operation of the slimming procedure.**

The code to do the slimming uses the OWLAPI library [22]. There are configuration files to specify the required information and the instructions for how the slimming should be performed (see https://github.com/enanomapper/slimmer/). For each imported ontology, a Java Properties file defines the input ontology, where the slimming instructions (i.e. what to include or exclude) can be found, and the IRI for the resulting slimmed OWL file. The slim files are created with ontology IRIs within the http://purl.enanomapper.net domain which was specifically set up for our ontology work. The IRIs for classes and properties are maintained as they are in the original input ontology. The pipeline operates as illustrated in Figure 1.

Firstly, the slimmer takes the configuration files, loads the primary ontology and merges it with the imported ontologies. This is to ensure that any class defined in any downstream imported ontology can also be exported into the slimmed ontology. During this process, all annotations on the ontology are maintained; these are complemented with annotation detailing the eNanoMapper project as publisher of this slimmed version of the source ontology, when the integrated ontology was made, and information about the tools used. The slimmer then removes all classes aside from those specifically listed for inclusion in the slimming instructions. The slimming instructions are defined in a separate file with a custom, dedicated format. Each line specifies an IRI of a class to keep, or to remove from a kept branch. For each IRI it is possible to further specify either that the chain of superclasses for that class, or all subclasses (i.e. a downward branch) should also be kept. This enables the selection of whole subtrees for inclusion, while still allowing for the possibility of pruning out components of the sub-tree individually. To ensure that the resulting slimmed files will arrange themselves hierarchically when used together in the composite whole, for each instruction it is possible to specify a superclass for the class with the given IRI, which superclass does not have to be defined in the ontology processed, i.e. this facility can link imported branches to components from other ontologies. Optionally, the line then terminates with a free-text comment which can be used to assist the human maintainers of the file, as the ontologies we have adopted do not use human-readable IRIs.

As an example, here is the current instruction file for the unit ontology (UO):

+D(http://purl.obolibrary.org/obo/IAO_0000030):

http://purl.obolibrary.org/obo/UO_0000000unit

This instruction says that the class UO_0000000 (unit) should be included from the unit ontology together with all its descendents, and that this class should be defined as a subclass of IAO_0000030 (information content entity), which in turn will be imported directly from IAO. The label of the class to be extracted is included only for human readability. While it is not necessary to specify a superclass for each IRI included in the instructions, it enables “cross-linking” imported classes or subsets to the remainder of the content in the assembled ontology without having to do repetitive manual work in order to stay up-to-date with the source ontologies as they evolve.

Using this slimming library we are able to build up our ontology from components drawn from existing ontologies. All of the generated slimmed ontologies are subsequently imported into a single OWL file, http://purl.enanomapper.net/onto/enanomapper-auto.owl which is in turn then combined with manually curated enanomapper content in the final ontology file http://purl.enanomapper.net/onto/enanomapper.owl.

In the next sections we describe which components we have selected from which external ontologies and how they have been interweaved.

### Nanoparticle types

Nanoparticles are typically classified primarily on the basis of their primary constituent, e.g. silica, carbon, titanium dioxide, gold or silver, nanoclay, etc., and their shape. The primary ontology relevant for nanoparticle types is the NPO. It was created out of the need to standardise data description in cancer nanotechnology research and enable searching and integration of diverse experimental reports. The NPO uses the Basic Formal Ontology (BFO, [23]) as upper level and is developed in the Web Ontology Language (OWL). It refers to multiple external ontologies including ChEBI. As of the last release (2011-12-08), the NPO contains 1,904 classes.

It includes the type of chemical components of a nanoparticle formulation which include the nanoparticle, active chemical constituents of the nanoparticle, and functionalizing components. The molecular structure of the chemical components is also included (e.g., atom, element, compound, liposome, micelle, etc.) – in some cases these have been imported from ChEBI.

ChEBI is both a chemical database and a chemical ontology. It offers a wide range of useful chemical information including chemical structures and properties, citations to the literature and both a structure-based and a role (activity)-based ontology classification. As of the last release (1 January 2015) there are 42,318 classes in ChEBI. The bulk of the chemicals annotated in ChEBI are organic molecular entities of biological interest as metabolites or agents that can intervene in biological processes. The most relevant for the eNanoMapper project are the functional groups and atoms which are used to describe the composition and functionalisation of nanoparticles.

The NPO contains an extensive classification of types of nanoparticle based on structure, function or chemical composition (e.g. ‘chitosan nanoparticle’ (NPO:261), ‘spherical nanoparticle’ (NPO:1551), ‘gold nanoparticle’ (NPO:401), ‘core-shell silica nanoparticle’ (NPO:1572), ‘fluorescent silica nanoparticle’ (NPO:1553) and ‘long circulating nanoparticle’ (NPO:1591)). ChEBI also contains a small ‘chemical substance’ branch with a small nanoparticle classification, primarily around types of nanoparticle based on their chemical constitution, some of which do not also appear in the NPO (thus are not superfluous). For example, ChEBI contains ‘palladium-gold nanoparticle’ (CHEBI:52523) which is defined as a gold nanoparticle covered with a thin coat of palladium atoms, and also ‘citrate-coated silver nanoparticle’ (CHEBI:82778).

We have imported both the nanoparticle classification from the NPO and from ChEBI, but for ChEBI we have removed those classes which duplicate classes in NPO.

Nanoparticles may be simple (e.g. nanodot with a particular composition) or complex, in that they may be composed of several layers and their surfaces may be heterogeneously functionalised with attached groups of any composition. We have imported from the NPO a few general classes for these complex particle types including ‘surface functionalized nanoparticle’ (NPO:1881); however, more specific detailed classes of types of functionalisation are not yet present. The molecular composition of the nanoparticle includes a specification of the constituent groups and atoms together with their bonding arrangement. When describing the molecular composition of nanoparticles it may be necessary to distinguish the molecular composition of specific parts of the nanoparticle, e.g. the surface, core, linkage etc. The NPO includes classes for the different parts of the nanoparticle e.g. ‘silica core’ (NPO:1865), and chemical linkages between chemical components (e.g., amide linkage, disulfide linkage, encapsulation) and the physical, chemical, or functional properties of chemical constituents and functionalizing agents (e.g., organic, hydrophilic, magnetic, etc.).

Nanoparticles are also commonly described by their dimensionality and shape. These aspects are partly covered by NPO. ‘Dimensionality’ describes the number of dimensions of the particle that are within the ‘nanoscale’ (i.e. between 1 and 100 nm). Thus quantum dots, hollow spheres and free nanoparticles, in which all three dimensions are in the nanoscale, are described as three-dimensional (some sources use the term ‘zero-dimensional’). Analogously, nanorods, nanotubes, nanowires and nanofibres, which have two dimensions in the nanoscale, are known as two-dimensional, while thin films or surface coatings, which have only one dimension in the nanoscale, are classed as one-dimensional. Nanoparticles come in different shapes, providing another useful descriptor for classification purposes. Thus two-dimensional nanoparticles may occur as rods, helices, zig-zags, or belts, whilst three-dimensional nanoparticles may be conical, cylindrical, ellipsoidal, elliptical, polyhedral, spherical, etc. Both the dimensionality and the shape of nanoparticles can be important factors in determining the toxicity of nanoparticles, their cellular uptake etc. In addition, nanoparticles have other relevant material or mechanical properties such as being soft or hard (stiff). The majority of these properties have been imported from NPO.

The NPO also contains items of relevance for bulk nanomaterial description, such as the physical state of a formulation (e.g., emulsion, hydrogel, etc.).

### ENM physico-chemical characterisation

The sorts of physicochemical properties that are used in the characterisation of nanomaterials include the state of dispersion, aggregation and agglomeration of the nanomaterial, the size (and size distribution) of the particle, the specific surface area and porosity, the surface composition and reactivity (a measure of the extent to which the surface atoms of the nanomaterial can induce the production of reactive oxygen species), and the purity (and impurities). Impurities can play a crucial role in determining the safety or toxicity of nanomaterials, so should be quantified and described.

The materials, rather than the particles, are described by the particle size distribution and the remainder of the medium in which the particles are contained, including solubility and dispersability in different media including water. Zeta potential is especially important to predicting the aggregation and agglomeration behavior of particles, and may be measured over a pH range. Diffusion and gravimetric deposition rates should be characterised, as they affect the dispersal and exposure time for substances leaking into the environment.

CHEMINF is an ontology of chemical information entities - descriptors and other chemically relevant data items, designed to support data sharing and standardisation of cheminformatics data in the context of the Semantic Web. As of the last release (5 December 2014), the ontology contains 732 classes. CHEMINF is already the standard for chemical property representation in Open PHACTS [24], and we have contributed a set of nanomaterial-relevant descriptors to it. Many cheminformatics descriptors included in CHEMINF, such as molecular mass, pKa and so on, are straightforwardly relevant also to data about nanomaterials, and we have imported the full branch of chemical descriptors into eNanoMapper from CHEMINF.

While not an ontology, the OECD Harmonized Templates are structured (XML) data formats for reporting safety-related studies [25] that contain vocabularies in the form of picklists for some of the specified fields, and documented guidance material. There are several templates that are specific to nanomaterial assessment over and above the templates that apply to all chemicals. These fall under the header “additional physico-chemical properties of nanomaterials" and include agglomeration/aggregation, crystalline phase, aspect ratio/shape, dustiness, porosity, pour density and radical formation potential. These terms, where not already present in NPO or CHEMINF, have been manually annotated in eNanoMapper.

### Biological characterisation

Through interactions with biological systems, nanoparticles may become covered with biological material, particularly proteins in the blood, and lipids in the pulmonary system. This is referred to as the “corona” of the nanoparticle and it strongly depends on the exposure medium (e.g. bovine serum) and the duration of exposure [26,27]. The corona may be composed of a monolayer or multiple layers, and the proteins may be denatured by their adsorption to the nanoparticle, creating entirely novel biomolecular entities with unknown reactivities. Similarly to protein-protein interactions, nanoparticle-protein interactions are characterised by binding affinity, stoichiometry, and kinetic properties.

The corona affects biodistribution and cellular uptake of the nanoparticle and may also cause some toxic effects. In short, exposure to any biological medium changes the external nature of the nanoparticle and thus its biological effects [28]. It is therefore very important that all data points include metadata to describe the history of each sample and to control carefully for exposure to biological material.

In addition to proteins, nanoparticles can also bind to and interact with DNA, or interact with whole cells [29]. Cell association (i.e. binding and uptake to cells of a given type) is a measurement of special importance for its relevance to inflammatory responses, biodistribution, and toxicity in vivo [27].

Foremost among the existing biological ontologies is the GO, the widely used ontologies for biological processes, molecular functions and cellular components used in gene product annotation. GO is the gold standard for annotation in these three sub-domain areas. It contains 42,329 classes (as of 22 December 2014).

For annotation of proteins, PRO may be used. With 83,656 classes (12 June 2014), it contains species-neutral grouping classes for all proteins identified in UniProt as well as links to the species-specific UniProt entries.

For annotation of cellular entities, the Cell (CL) ontology may be used (6,287 classes as of the 22 December 2014 release). The Cell Culture Ontology is also relevant (CCONT).

Importing biological entities from external ontologies is common practice in assay-related ontologies. For example, BAO has a good selection of imported biological entities for use in annotation of assays against biological endpoints.

The NPO also includes content of relevance for the biological characterisation of nanoparticles, including the underlying mechanisms guiding the design for a particular formulation (e.g., endocytosis, active targeting, etc.), types of stimuli (e.g., magnetic field, ultrasound, pH change, etc.) for activating the function of nanoparticles, and the responses to those stimuli (e.g., drug release from nanoparticle in response to magnetic field, heat generation from nanoparticle in response to infrared light, etc.), the biochemical roles or functions of included chemical components (e.g., anticancer drug, surface modifying agent, MRI contrast agent, spacer, etc.) and applications of nanomaterials especially in cancer diagnosis, therapy, and treatment (e.g., chemotherapy, diagnostic imaging, detection of cancer cells, etc.).

### Environmental characterisation

Nanoparticles may be released into the environment throughout their lifecycle, including their initial synthesis, incorporation into a product, use by consumers, and disposal, so subjecting workers, consumers and the environment to potential exposure. A variety of methods and measurements may be used in order to assess exposure, including the use of particle number, particle mass, and surface area detection devices.

When describing the environmental hazards and assessments of the environmental impact of nanomaterials, once they get ‘into the wild’, it will also be necessary to refer to a wide range of different ecosystems, environment types and locations.

ENVO contains terminology covering a wide range of environments including, for example, marine zones, tidal zones, soil and so on (1,691 classes as of the 17 September 2014 release). It also contains biomes such as desert and grassland; environmental features such as archaeological sites, caves and beaches; and environmental conditions such as arid and subtropical.

### Experimental measurements and protocols

In addition to the various measurement outcomes (physicochemical and biological characterisation and properties) discussed above, measurement techniques and tools are included in the eNanoMapper ontology. These include [30]:

- Transmission electron microscopy, scanning electron microscopy and atomic force microscopy provide information about the nanoparticle morphology.
- Crystallographic methods can be used to determine the shapes of particles.
- Dynamic light scattering (DLS) provides information on the hydrodynamic radii of nanoparticles in solution.
- Surface charge properties are determined with zeta-potential measurements.
- Chemical composition is revealed by auger electron spectroscopy, x-ray photoelectron spectroscopy, time-of-flight mass spectrometry and elemental analyses.
- Surface ligands and adsorbed molecules are identified with magic angle spinning nuclear magnetic resonance, liquid chromatography mass spectroscopy (LC-MS) and Fourier-transform infrared spectroscopy. Surface-enhanced Raman spectroscopy may also be used.
- Size exclusion or thin layer chromatography can identify nanoparticle-bound lipid molecules.
- Binding of surfactant molecules onto the surfaces of nanoparticles may alter their surface plasma resonance absorption and can also be determined using UV-vis absorption spectroscopy.
- Surface pressure-area isotherm measurements can be used to study the properties of lipid monolayers in the presence of nanoparticles.
- Differential scanning calorimetry (DSC) and isothermal titration calorimetry (ITC) can be used to measure thermodynamic changes in supported membranes or liposomes.
- Steady-state and time-resolved fluorescence spectroscopy are used to study nanoparticle-protein binding affinities, complex formation, and binding-induced protein conformational changes.
- Stepwise photobleaching has also been used to characterize nanoparticle-protein interactions.
- Proteins bound to a nanoparticle surface may be identified by 2D polyacrylamide gel electrophoresis.
- The adsorption and desorption processes of nanoparticle-DNA complexes can be measured using cyclic voltammetry.
- Cellular uptake can be monitored using X-ray fluorescence microscopy to determine the chemical element distribution of nanoparticles in cells.
- Magneto-photoacoustic imaging can be used to differentiate membrane-adhered from endocytosed nanoparticles in a cell.
- Atomic force microscopy measures the force between nanoparticles and the cell surface in cell association.

The details of the experimental methods captured in an ontology may include links to instruments used in the measurements. The NPO contains experiment types of relevance in nanomaterial characterisation, e.g. ‘dynamic light scattering’; and experimental methods for synthesis, e.g. ‘solvent displacement method’. More experimental types of relevance for physicochemical characterisation are also included in CHMO and REX.

ISA-TAB is a commonly used format for representing experimental data in structured tab-separated files [31]. There are three file types: investigation, study and assay. The investigation file contains the broad reference information about the project in the context of which the biological experiment has been performed, such as the point of contact and publications, and includes reference details for the other files. The study file includes all the information about the sample being tested, and the investigation file records the raw data of the assay (or specifies the files for the raw data for non-spreadsheet, e.g. image data). The ISA-TAB Nano specification enhances the “pure” ISA-TAB specification with support, in the form of an additional file type “material”, for describing nanomaterials [32]. While the study file enables description of samples of biological origin, the material file enables description of samples of non-biological origin, whether nanoscale or not. It enables the description of complex nanomaterial formulations including chemical components, functionalizing agents, and medium of suspension.

There are at least two ontologies in the biomedical domain that include biological assay descriptions: OBI and BAO. While OBI is broader in scope and has the involvement of many different communities in its development, BAO has arisen from a more specific targeted need in chemical biology data annotation. OBI has 2,799 classes as of the last release (17 December 2014) and BAO has 3,340 (11 November 2014). As will be discussed further below, OBI is more metadata-rich than BAO, while BAO has a neater classification hierarchy. OBI also has a wider diversity of assays represented. However, their content is not fully overlapping and both are relevant (thus we have imported assays from both). Neither OBI nor BAO have any nanomaterial-specific content or assay types.

The explicit and detailed protocols for all assays and measurements should ideally be captured in ontology annotations in just as much detail as they are described in the experimental methods sections of high-quality publications, i.e. in sufficient detail to allow them to be reproduced. The ontology, however, can only supply the vocabulary to be used in such descriptions. The enforcement of the minimum level of detail required when annotating data of a given type (about a particular experiment) needs to be done via alternative methods combined with the use of the ontology as knowledgebase and vocabulary. For example, experimental templates such as the OECD Harmonized Templates can suggest which fields need to be filled for various different types of experiment, and Minimum Information guidelines could be created to use as checklists for automatic quality-checking of data. Core content checklists such as the OECD HTs have previously been formalised for this purpose in the context of the OpenTox project [33,34]. To this end, in addition to the dedicated annotation of assays captured in ontologies, the PROV-O ontology (a W3C recommendation, [35]) provides many useful classes and relationships for expressing provenance information associated with data, which can include who generated it, when, what it is derived from, and what software was used in the production.

### Nanomaterial lifecycle

The full ‘cradle to grave’ nanomaterial lifecycle from synthesis through use to recycling, disintegration or environmental accumulation needs to be described in the ontology.

While coverage of this aspect of nanomaterials is poor in existing ontologies, the NPO contains a few relevant classes, including ‘biodegradable nanoparticle’ (NPO:836) as a class of nanoparticle type. The definition in NPO refers to the property (quality) of being ‘biodegradable’ (NPO:191). NPO also contains a small number of manufacturing-relevant classes under ‘material synthesis technique’ (NPO:1921).

For nanomaterial manufacturing, the InterNano Nano-Manufacturing Taxonomy provides vocabulary, using numeric identifiers. It is available at http://purl.bioontology.org/ontology/InterNano. It includes branches such as application areas for nanotechnology, health and safety, nanomanufacturing and characterisation processes, a classification of nanomaterial types, and social and economic impacts. The taxonomy is used for intelligent searching and to organize content on the internano.org website. While a very useful resource in terms of terminology, the taxonomy does not include additional metadata, such as synonyms or definitions. It also does not include further ontological content such as relationships (other than hierarchy).

### Known safety information

The ontology should support the rapid retrieval of relevant safety information given a particular class of ENMs and a particular biological context. While the safety data itself will be included in the eNanoMapper database (not the ontology), the ontology needs to include classes for different types of toxicological endpoints as well as the experiments that are conducted to evaluate and assess toxicity in different systems.

Regulatory language used to describe safety hazard classes is also required in the context of the ontology, to enable organising and searching the known information from the literature.

In order to describe safety outcomes in medical contexts we have incorporated the classification of adverse events from the Ontology of Adverse Events (OAE, [18]) which comprehensively annotates adverse events or reactions of medical relevance. An example of an adverse event from this ontology is ‘hypertension adverse event’ (OAE_0000403).

### Summary

**Figure 2:**
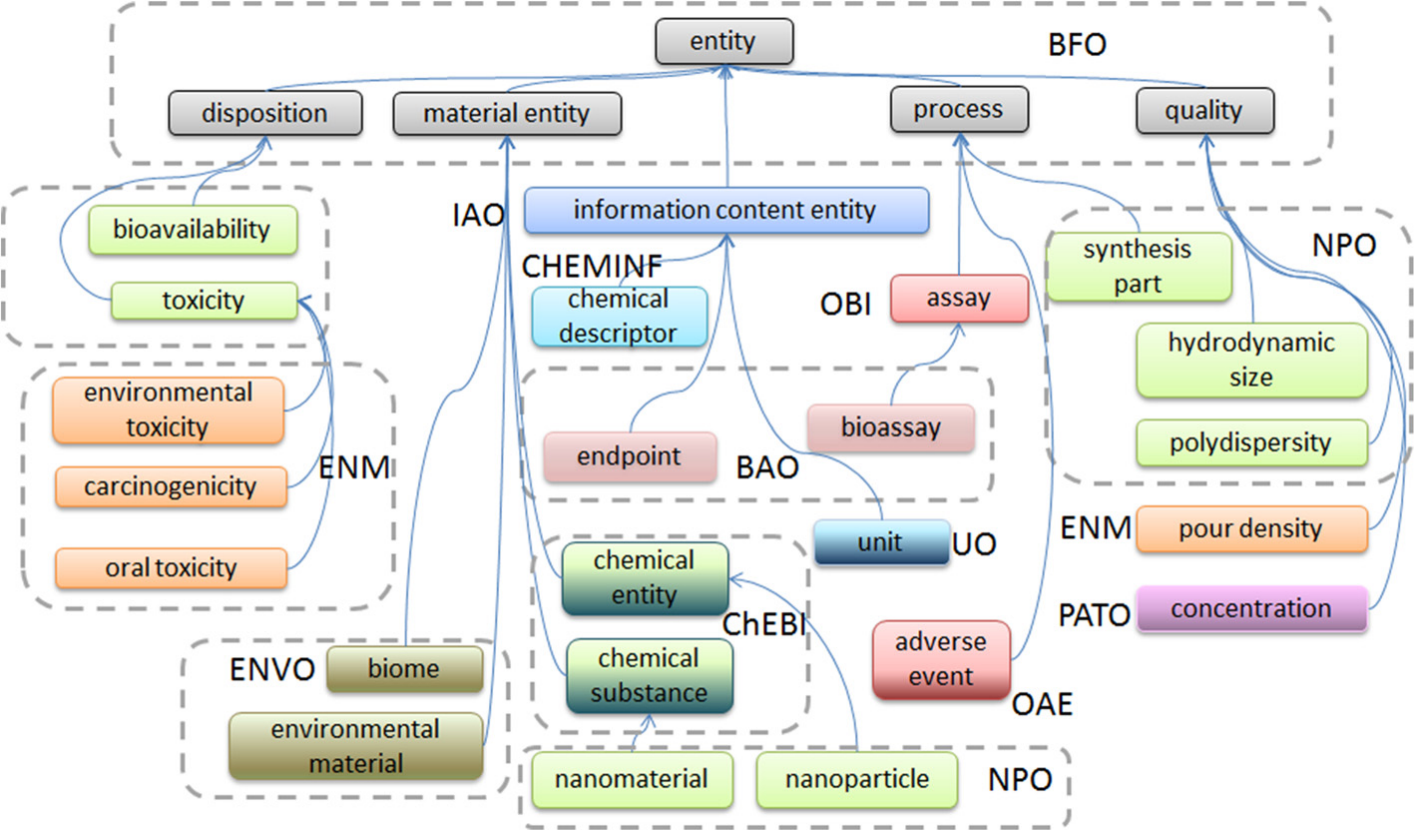
**An overview of the upper levels and integration of external ontology content together with manually annotated (ENM) content.**

The different components that have been discussed above have been integrated into a composite ontology in which the original IRIs and hierarchical structure for the subsets which are imported are maintained, but the way they are assembled together is through specifying new parents for imported subset modules such that they automatically fit together. A subset of the resulting ontology is illustrated in Figure 2, with an emphasis on how the slimline upper level (a subset of BFO) is used to ‘glue’ together the different parts of the external ontologies in a coherent fashion. Some ontologies have components which form subsets in multiple different basic types (such as the NPO, BAO), while others mainly contribute their content to one branch or to one main content type. Our manually annotated content can also be observed in multiple different branches (tagged as ENM), extending classes imported from elsewhere.

As part of the overall eNanoMapper project, a database is being developed in which the content will be richly annotated with ontology IDs [36]. To assist researchers in providing such annotations, an ontology annotation recommendation tool is being developed that wraps the NCBO annotation recommendation service [37] in the R language (https://github.com/muntisa/RNCBO). It takes as input a list of terms needing annotations, and as output suggests possible annotation values based on the lookup across the BioPortal collection [38]. The challenges with overlaps and gaps in content described here also apply to this tool as long as it harnesses existing publicly available content. In the future, this recommendation tool will be extended to offer a version that has been restricted to the eNanoMapper ontology specifically, in which we aim to address these challenges.

## Discussion

### Coverage

Each of the ontologies mentioned in the above content areas provides partial to good coverage of their specific domain areas. However, the composite suite of external ontologies nevertheless does not cover the full domain of nanosafety as needed for eNanoMapper. Further experiments need to be annotated, particularly toxicology and nanomaterial-specific experiments, and nanomaterial-specific properties need to be added to various ontologies. Even dedicated ontologies such as the NPO need to be updated in the light of recent advances in the field of nanotechnology safety, including a growing diversity of novel nanomaterial types and new approaches to describing biological interactions.

In an effort to concretely evaluate the coverage of the ontologies against the terminological annotation requirements arising from public data, we used two targeted data annotation terminology sets. These were the characterisation properties described in the “protein corona” dataset (the data from [39]) in the eNanoMapper prototype database ([36]) and the terminology used in the OECD Harmonized Templates specific to nanomaterial characterisation.

From the protein corona dataset characterisation properties, of 21 properties reported 12 had exact matches within the external ontologies being imported, while an additional five had close matches where a composite annotation would serve the purpose. Examples of terms that had exact matches include DLS (NPO_1469), polydispersity index (NPO_1155) and *g*/*cm*^3^ (NPO_1270, UO:0000084). Partial matches include ‘z-average hydrodynamic diameter’ which can be annotated with a composite of NPO_1916 (z-average) and NPO_1915 (hydrodynamic diameter). Of the terms that had no match at all included ‘per NP’ units such as *cm*^2^/*NP*. These are nanomaterial-specific units that are in scope for submission to the NPO.

Of the 26 terms in the OECD HTs dedicated to nanomaterial characterisation, six had no exact match in the imported ontology suite, including ‘dustiness’, a property that can be defined as the propensity of a powder to become aerosolised by mechanical agitation, dependent on its moisture content and static electrical properties [40], ‘pour density’, and ‘crystallite and grain size’. We have annotated these in the manual eNanoMapper content, and they are within scope for, thus will be submitted to, the NPO. There were also many terms in the OECD HTs that were not specific to nanomaterials but rather to evaluations of the safety of any chemical, which were missing from our suite of imported ontologies. These include classes for biodegradation under different circumstances, e.g. ‘biodegradation in water’, ‘biodegradation in soil’, and ‘bioaccumulation’. It also includes many different toxicity classes such as ‘short-term toxicity to fish’, ‘acute toxicity - inhalation’ and ‘teratogenicity’. Some of these toxicity classes are present in the OpenTox endpoint ontology that was developed in the context of the OpenTox project. However, this ontology is not interoperable with the remainder of the ontologies in the suite as it does not use numeric identifiers complemented with textual labels and definitions. Thus, the content of the OpenTox endpoints ontology as well as the other toxicology-related terms not yet included have been added to an endpoints branch of the eNanoMapper ontology manually curated content as subclasses of BAO’s endpoint class, http://purl.enanomapper.net/onto/internal/endpoints.owl.

In general, nanotechnology-specific assays, properties and related materials are sparsely represented in existing ontologies and thus need to be added to the existing assay and chemical information ontologies. In order to be of the widest possible benefit, the eNanoMapper project envisions submitting relevant terminology to targeted external ontologies for inclusion at the source rather than appending content in the eNanoMapper ontology only. Much of the missing content that we have identified so far is in scope for one of the ontologies already targeted for import, e.g. the NPO, BAO or OBI, CHEMINF, ChEBI or CHMO.

Several of the content areas that are relevant for nanomaterial safety assessment are thus far not covered by any existing publicly available ontology, thus will need to be manually annotated into eNanoMapper. One such gap is the nanomaterial lifecycle, from manufacturing through to environmental and biological impact. These toxicology and safety-related terminology areas present a gap which is not presently covered by an ontology that conforms to the OBO Foundry [41] recommendations for interoperable ontologies. The InterNano Nanomanufacturing Taxonomy forms a good starting point for terminology in this area, but that terminology needs to be defined and further annotated. Known safety information is another gap. Database efforts such as the OECD Database on Research into the safety of manufactured nanomaterials may serve as a starting point for this.

### Harmonizing definitions

Ontologies contain additional information relative to the labels and identifiers that are so useful for annotation. They contain definitions, relationships and complex axiomatisations in terms of those relationships. The NPO already contains a sophisticated axiomatisation for some parts of the ontology, embedding detailed domain knowledge of relevance for nanomaterial research into logical definitions captured in the ontology. This is described in detail in [5].

For example:

- the class ‘intracellular fluid in a tumor cell’ is defined as “ ‘intracellular fluid’ and (contained_in some ‘intracellular space of tumor cell’) and (has_quality some ‘tumor intracellular pH’)”;
- the class ‘carbohydrate-coated nanoparticle’ is defined as “nanoparticle and (has_component_part some (carbohydrate and (has_role some “nanoparticle surface modifying role’)))”;
- the class ‘fluorescence imaging contrast agent’ is defined as “ ‘optical imaging contrast agent’ and (has_application some ‘fluorescence imaging’) and (has_property some fluorescent)”;
- the class ‘elimination rate constant’ is linked to the class ‘pharmacokinetics study’ using the relationship ‘parameter determined from’.

However, such axiomatisations can differ remarkably from ontology to ontology, a fact not always highlighted in a straightforward overlap analysis. For example, the definitions for the assay classes in OBI and BAO are very different. OBI defines ‘assay’ as

“A planned process with the objective to produce information about the material entity that is the evaluant, by physically examining it or its proxies.”

BAO, on the other hand, does not include ‘assay’ as a term, it includes ‘bioassay’, defined as

“A set of instructions, methodology, operations, required reagents, instruments to carrie out experiments for the purpose of testing the effect of a perturbing agent in a biological model system, measuring one or multiple effect(s) of the agent facilitated by an assay design method translate the perturbation into a detectable signal to arrive at one or multiple endpoint(s) that quantify or qualify the extent of the perturbation. Bioassay is described by multiple bioassay components: assay format, biology (biological participants in various role and processes), design method, physical detection method/technology, screened entity, and endpoint. Bioassay includes one or multiple measure groups to describe panel, profiling, multiparametric (or multiplexed) assays (assays that measure more than one effect of the perturbagen on the system that is screened)”.

**Figure 3:**
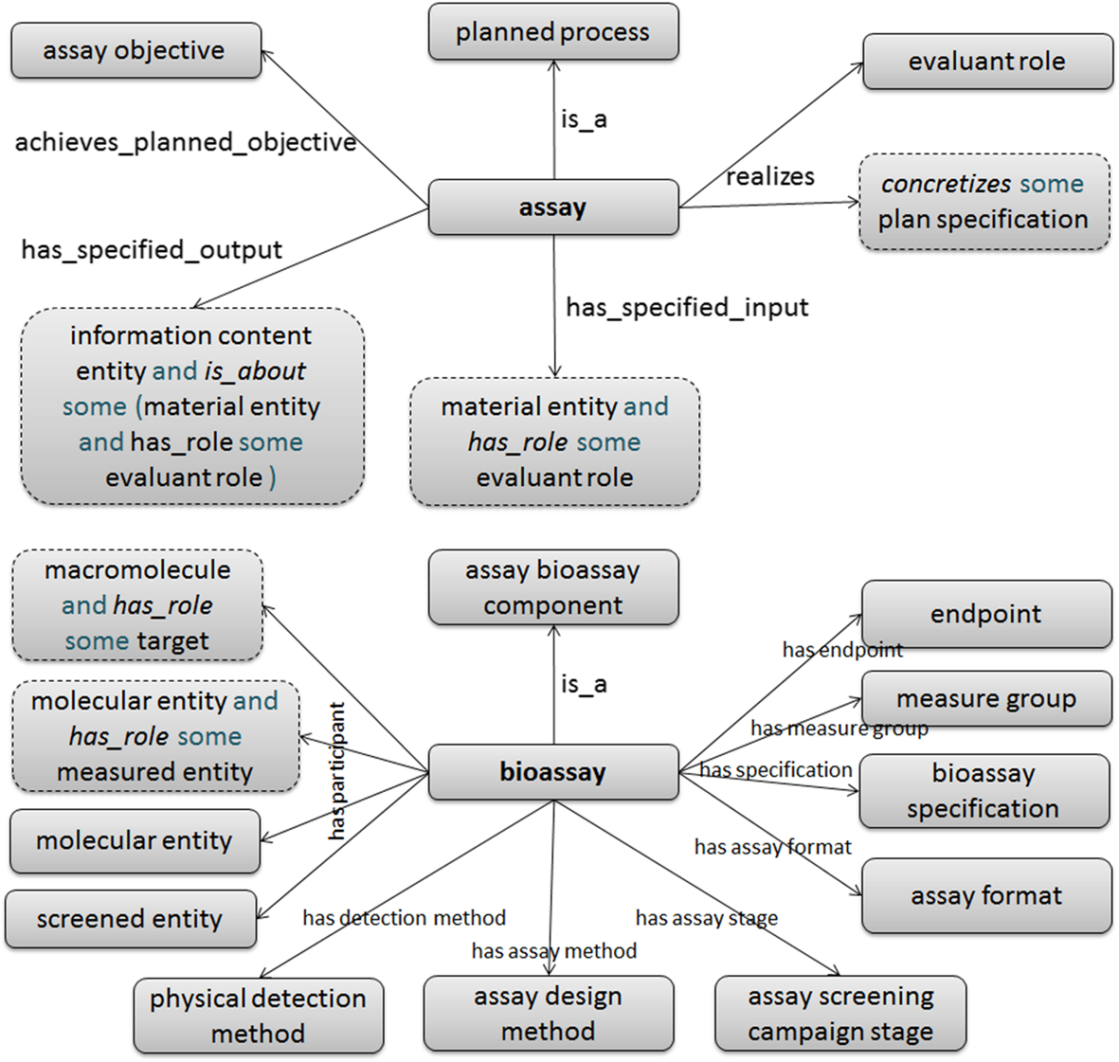
**The axioms associated with the core ‘assay’ entity in OBI and ‘bioassay’ in BAO.**

From these textual definitions, one might infer that these two ontologies ostensibly covering the same domain area have radically different conceptualisations of the content. This is also evidenced in their differing axiomatisation for the terms. As can be seen in Figure 3, OBI has a smaller model using generic (not domain-specific) relationships such as ‘realizes’ and ‘has role’, while BAO has a plethora of bioassay-specific relationships linking the core assay entity to many different sorts of component e.g. detection method, endpoint and measure group. On the other hand, while BAO explicitly marks these semantically distinct aspects with their own relationships and hierarchies, OBI includes this information in the assay classification hierarchy (e.g. ‘flow cytometry assay’ is a type of assay). And indeed, OBI contains a significantly larger number of different assay classes beneath their ‘assay’ entity than BAO does.

**Figure 4:**
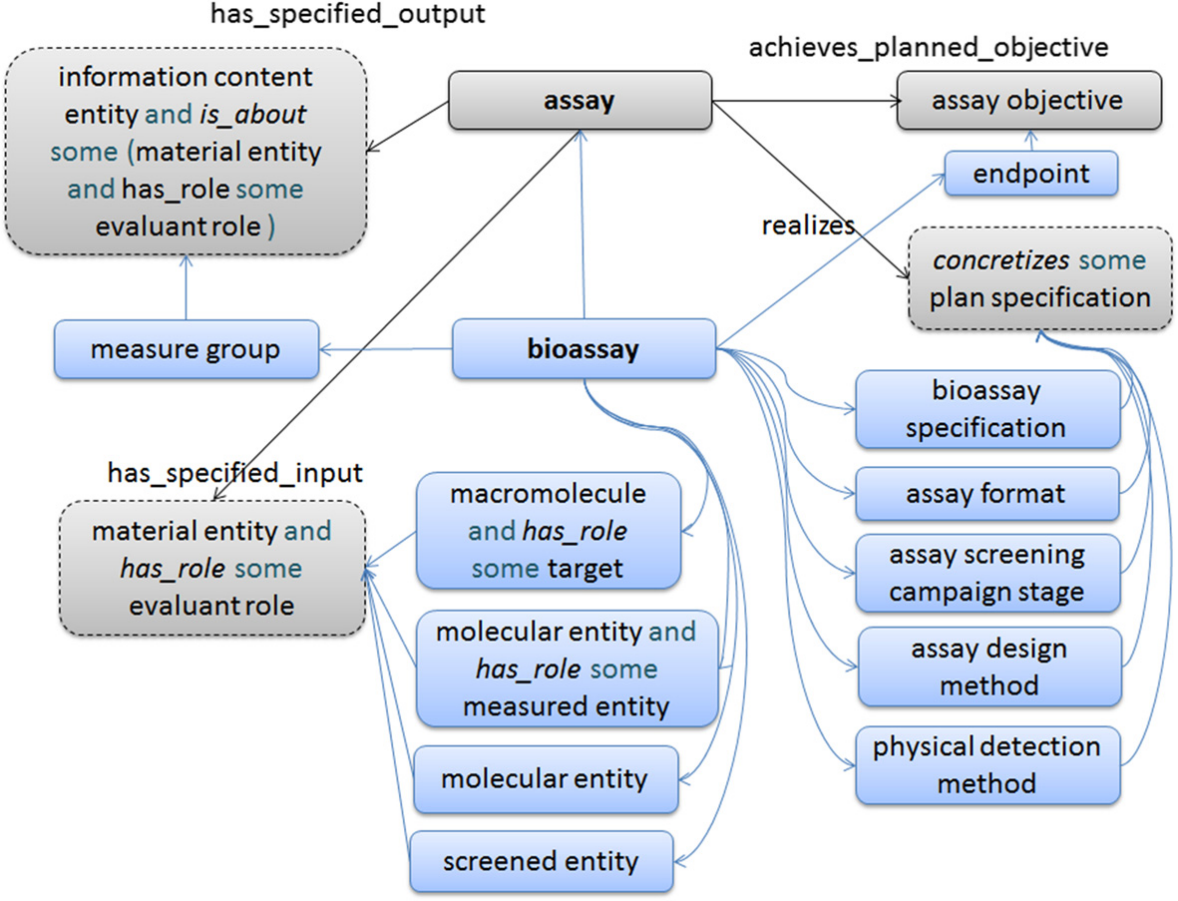
**Relating ‘assay’ from OBI with ‘bioassay’ from BAO.** Entities from OBI are shown in grey while those from BAO are blue.

However, considering that both ontologies reflect the same underlying processes (i.e. experiments), it is possible to broadly synthesise their models, identifying appropriate mappings between the classes and relationships used in the two implementations. This integration is illustrated in Figure 4.

The integration consists of the following mappings:

- **Upper level:** BAO does not present a standard upper level classification, while OBI is classified beneath BFO 1.1. Since eNanoMapper has adopted a slim upper level drawn from a subset of BFO 2.0., we have mapped OBI’s ‘assay’ class to BFO’s ‘process’ in our subset, and furthermore mapped BAO’s ‘bioassay’ class to OBI’s ‘assay’ as a parent, bringing both hierarchies into the same branch of the ontology.
- **Assay specification:** OBI uses two constructs to capture the design and objective of each assay: the ‘assay objective’ and the ‘plan specification’. BAO, on the other hand, uses several different classes to capture specific aspects of the design and objective of the assay: the classes ‘physical detection method’, ‘assay design method’, ‘assay screening campaign stage’, ‘assay format’, ‘bioassay specification’ and ‘endpoint’ all capture aspects of these areas. Thus, we relate the OBI classes to those of BAO as illustrated.
- **Input:** Assays typically have various subtances containing e.g. molecular entities, proteins and so on as participants. In OBI this is captured as a specified input of a ‘material entity’ – i.e. very broad so as to allow for all the possible different types of assay beneath the same umbrella. In BAO, on the other hand, the different types of input are listed specifically as a ‘screened entity’, ‘molecular entity’ as ‘measured entity’ and ‘macromolecule’ as ‘target’. Again, here we can subsume the BAO entities beneath the OBI more broad axiomatization.
- **Output:** BAO captures the measured output of an assay as a ‘measure group’, which in turn is a holder for a table-like grouping of different outcomes or measurements. OBI on the other hand captures specifically that the specified output of an assay is an ‘information entity’. This can be tied together by classifying BAO’s ‘measure group’ beneath the generic IAO class ‘information content entity’.

The PROV-O model for provenance of information is also related to the assay models of BAO and OBI. At the core level, prov’s ‘activity’ class broadly subsumes the OBI ‘assay’ class. PROV-O has an explicit representation of ‘agent’, which in the context of the assay can be taken to be the scientist who is performing the experiment, which can be represented in OBI and BAO at the level of the responsible organisation. Another straightforward correspondence is to the PROV-O’s ‘plan’ class, which is related to the classes ‘plan specification’ and ‘bioassay specification’ in OBI and BAO respectively. Many of the relationships included in PROV-O are useful for incorporating additional metadata about assays and the information entities derived from them.

The challenge of conflicting definitions is well known in the nanomaterial domain, with the community as a whole struggling to come up with a definition for the entities that are its focus. The NPO defines a nanomaterial as: “A chemical substance which has at least one external dimension or internal structure or surface structure in the nanoscale size range”, and nanoparticle as: “A primary particle which has an average size in the nanoscale range; which has an identifiable and definite chemical composition, property or function that uniquely define the nanoparticle’s type as known; and, which may or may not exhibit a size-intensive property”. NPO has drawn on community standards for these definitions, and given that NPO is the primary ontology for nanomaterial description, ideally we would like to adopt their definitions. However, from an ontological perspective the definitions could be said to show some confusion. Firstly, it is not clear from these definitions alone what the distinction is between nanomaterial and nanoparticle. We might assume that the nanomaterial is an aggregate consisting of an arbitrarily large number of nanoparticles of a given type (i.e. bulk granularity in the terminology of [42]), and that the nanoscale size range referred to would be the size of the components of the aggregate rather than the material as a whole, which components are then the nanoparticles. Given this assumption, it is perplexing that the given nanoparticle definition refers to an average size, when a single particle clearly cannot have an average size. Secondly, strictly speaking, the final clause ‘may or may not exhibit a size-intensive property’ adds no information, however, from an understanding of the domain one may gather that this clause is there because often nanomaterials do display size-specific properties, and that is precisely why there is so much interest in them. Thus this addendum might be better included as a comment. A straightforward definition of nanomaterials might say that they are materials containing structures in the approximately 1-100 nm scale which exhibit novel properties because of their small scale. The EU definition along these lines removes the term “approximately”, in order to allow its usage in a legal context, resulting in the following definition of nanomaterial:

“A natural, incidental or manufactured material containing particles, in an unbound state or as an aggregate or as an agglomerate and where, for 50 % or more of the particles in the number size distribution, one or more external dimensions is in the size range 1 nm - 100 nm. In specific cases and where warranted by concerns for the environment, health, safety or competitiveness the number size distribution threshold of 50 % may be replaced by a threshold between 1 and 50 %” [43].

This definition has been criticised on scientific grounds related to the potential limitation involved in defining strict bounds for the size, distribution and agglomeration. There has even been controversy about whether a definition for nanomaterials should be adopted at all, with [1] arguing against on the grounds of heterogeneity in the domain (e.g. different size ranges confer reactivity on different basic chemical structures), while [2] countering that despite the heterogeneity, a definition that stands up to legal scrutiny is essential in creating a regulatory environment that is able to protect the consumer from otherwise invisible hazards (e.g. by enforced labelling for this class of materials in consumer products). Leaving regulatory considerations aside, on a practical level for the eNanoMapper project these classes should not remain undefined, to comply with ontology best practices and in support of data annotation: given the above similarity in definition between NPO’s ‘nanomaterial’ and ‘nanoparticle’, it might be difficult for data providers to decide which term to use to annotate their data. It should also be noted that a definition based only on the size of the individual components of the aggregate material alone potentially also includes many types of molecules that do fall within the relevant size but which are not normally components of nanomaterials. Here, the distinction between engineered molecular entities and those which are naturally occurring is relevant, but this distinction is not reflected in any of the available definitions.

In order to address this definitional challenge within eNanoMapper, we have explicitly differentiated the ‘nanomaterial’ class from the ‘nanoparticle’ class by following ChEBI’s distinction between ‘chemical substance’ and ‘molecular entity’. This means that we have defined a material as some portion of a bulk aggregate substance, and a particle as a single individual within such an aggregate. This necessitates being careful to annotate particle size distribution as a property of the material rather than as a property of the particle.

## Conclusion

We have presented the development and current structure of eNanoMapper, an ontology which draws heavily on existing ontologies in order to provide a unified ontology for the annotation of data about nanomaterial safety. Re-use of ontology content in the development of new ontologies is not novel, and indeed is considered good practice by ontology organisations such as the OBO Foundry [41]. However, the manual inclusion of content from third-party ontologies is very difficult to maintain in the longer term, even if the MIREOT approach is used [19]. We have thus presented a library to facilitate ontology re-use through extracting subsets of existing ontologies according to a small but versatile set of instructions, which allows the resulting branches and components of different ontologies to be automatically pieced together. The closest existing tool for a similar application is the OntoFox tool [44], which uses SPARQL to extract subsets from existing ontologies, by default offering the OBO Foundry collection of ontologies as sources. Compared to OntoFox, our solution offers no GUI or web service interface, but rather is designed to run as a library in a fully automated pipeline. Furthermore, OntoFox does not allow the specification of new parents for the imported modules such that the module fits into an integrated whole in a fully automated fashion.

Since the domain of nanomaterial safety includes such broad sub-domains as biological experiments, physicochemical and environmental characterization, and molecular and biological entities, it would be an enormous effort to redevelop such an ontology “from scratch”. Re-use of already existing resources is not only less wasteful, it is in fact the only way to ensure that the ontology is able to be developed in the limited time frame available to the eNanoMapper project. The type of integration which we have attempted is, however, uniquely challenging and involves both structural and conceptual synthesis activities, which is particularly evident in the case of the integration of the various assay classes from different sources (BAO, OBI, NPO). We have presented our first steps towards a comprehensive integration of all content relevant to this challenging domain, and described both manual and automatically assembled content in the eNanoMapper ontology.
